# The Impact of Cobalt Substitution on the Electrochemical Properties of Cubic Li_6.6_La_3_Zr_1.6_Ta_0.4_O_12_ Solid Electrolyte for All‐Solid‐State Li‐Ion Batteries

**DOI:** 10.1002/advs.78649

**Published:** 2026-11-09

**Authors:** Jehad Ahmed, Jordan A. Barr, Zhizhen Qin, Anna Domgans, Kwangnam Kim, Chih‐Long Tsai, Roland Schierholz, Julian Borowec, Brandon C. Wood, Liwen F. Wan, Anna Windmüller, Florian Hausen, Rüdiger Eichel

**Affiliations:** ^1^ Institute of Energy Technologies: Fundamental Electrochemistry (IET‐1) Forschungszentrum Jülich GmbH Jülich Germany; ^2^ Institute of Physical Chemistry RWTH Aachen University Aachen Germany; ^3^ School of Mechanical and Material Engineering Washington State University Pullman Washington USA; ^4^ Laboratory for Energy Applications for the Future (LEAF) Lawrence Livermore National Laboratory Livermore California USA

**Keywords:** composite cathode, microstructure, side reaction, sintering atmosphere, solid‐state lithium‐ion battery

## Abstract

Garnet‐type Li_7_La_3_Zr_2_O_12_ (LLZO) is a promising solid electrolyte for all‐solid‐state lithium batteries (SSLB). The diffusion of cobalt (Co) into LLZO in combination with LiCoO_2_ (LCO) as the active cathode material (CAM) is widely reported in FAST/SPS‐sintered LCO/LLZO composite cathodes, sintered under an argon (Ar) atmosphere, and associated with degradation. In contrast, air‐sintered LCO/LLZO composite cathodes often demonstrate greater cycle stability, indicating an impact of the processing atmosphere. To address this issue, this study systematically investigates the effect of Co substitution in Li_6.6_La_3_Zr_1.6_Ta_0.4_O_12_ (LLZTO) sintered under both atmospheres. The structural, microstructural, and electrochemical properties of the sintered materials were characterized using XRD, SEM‐EDS, Raman spectroscopy, and EIS. DFT calculations were further performed to investigate the thermodynamic stability of Co substitution at different cation sites within the LLZO structure. Our results demonstrate that Co incorporation into the LLZTO lattice is significantly greater under Ar, with Co^2+^ occupying Li‐positions according to DFT, and total conductivity is reduced. When sintered in air, most of the Co segregated as a secondary phase of LCO and electronic conductivity is increased. These results identify one possible contribution to the degradation of LCO/LLZTO composite cathodes processed under reducing conditions and suggest that post‐annealing in air may reverse it.

## Graphical abstract

Cobalt is incorporated more readily into garnet Li_6.6_La_3_Z_r1.6_Ta_0.4_O_12_ when sintered in argon than in air. Under argon, Co in the garnet lattice lowers the total conductivity three‐ to fourfold; in air, Co segregates as LiCoO_2_ and raises the electronic conductivity about 100‐fold instead. These findings may explain why LiCoO_2_/Li_6.6_La_3_Z_r1.6_Ta_0.4_O_12_ cathodes processed under reducing conditions require a post‐annealing in air.

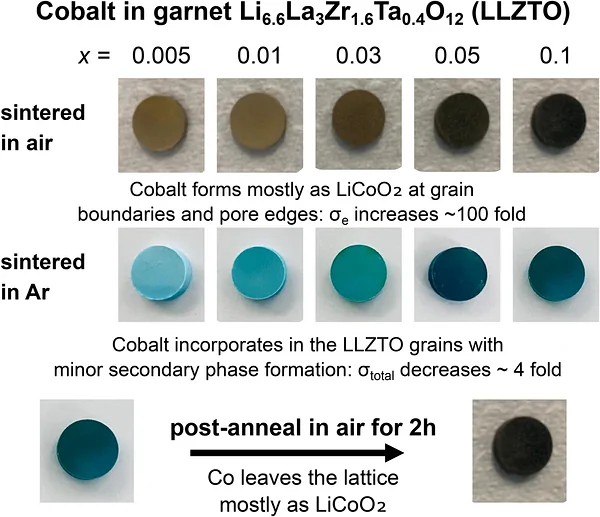

## 1 Introduction

Modern energy storage systems, especially with the state‐of‐the‐art Lithium (Li)‐ion batteries (LIBs), are crucial for the development of electric vehicles (EV) and large‐scale stationary renewable energy storage. Nevertheless, the requirements for higher energy density for extending EVs’ driving distances and stationary energy storage operations, higher safety to avoid fire hazards during battery operations, faster dis/charging allowing high power output or shortening charging times, and longer lifespan for lowering the overall usage costs cannot be fulfilled by today's LIBs [1]. SSLBs, which incorporate solid electrolytes instead of liquid ones, offer significant advantages over conventional Li‐ion batteries (LIBs). This is due to their potential to achieve higher energy and power density by using Li‐metal as the anode instead of intercalation graphite, as well as offering higher safety by eliminating the risk of flammable organic liquid electrolytes. They are also more environmentally friendly, eliminating the risk of toxic liquid electrolyte leakage [2, 3, 4, 5].

Among recently discovered solid electrolytes (SEs), garnet‐structured Li_7_La_3_Zr_2_O_12_ (LLZO)‐based materials have been considered as one of the promising SEs for SSLB applications. LLZO possesses several attractive properties such as high ionic conductivity in the order of 10^−4^–10^−3^ S cm^−1^ at room temperature, low electronic conductivity, a wide electrochemical stability window including stability toward metallic Li, good chemical stability in ambient atmosphere, and very high elastic property [6]. Specifically, the Ta‐substituted LLZO (LLZTO) shows high compatibility with Li‐metal, which enables the direct integration of Li‐metal [7]. Furthermore, the very high elastic property of LLZO allows this type of SSLBs to operate without applying high external pressure, as they differ from sulfide‐ and halide‐type SSLBs, which provides the potential for achieving higher energy density and safety at the pack level due to no need for an external pressure management system [8, 9]. Nevertheless, the very high elastic property of LLZO, which usually needs a high‐temperature sintering process for establishing ionic and electronic conductive paths in the microstructure, also brings the disadvantage of using it in SSLB fabrication, as the electrolyte cannot accommodate the volume change of the cathode active material in composite cathodes [8, 9, 10, 11, 12].

The development of LLZO‐based SSLBs until now can be classified into two different approaches: (1) liquid phase sintering by using a sintering additive [9, 13, 14]. and (2) direct co‐sintering [8, 10, 11, 12]. Both approaches use LiCoO_2_ (LCO) as their active cathode material due to its high thermal stability toward LLZO, similar thermal expansion coefficients, and high electronic conductivity [10, 13, 15, 16]. Han et al. demonstrated that Li_2.3‐x_C_0.7+x_B_0.3‐x_O_3_ (LCBO) can effectively establish the ionic and electronic diffusion paths within the composite cathode, i.e., LCO/LLZTO/LCBO mixture, and to the LLZTO solid electrolyte at 700°C [14]. The SSLB demonstrated stable cycle performance for 100 cycles at 25°C. Nevertheless, the very low ionic conductivity of LCBO (≈ 10^−6^ S cm^−1^ at room temperature) raises the concern of being able to provide an SSLB with a higher specific areal capacity than demonstrated [17]. Recently, Yamada et al. proposed the use of LiOH as the sintering additive for liquid phase sintering [9]. Although the final optimized sintering temperature still reached 900°C, they demonstrated that their SSLB with LCO/Ca‐substituted LLZTO/LiOH composite was able to reach an initial discharge specific areal capacity of 1 mAh cm^−2^ (8.1 mg LCO loading) at 25°C and 46% capacity retention after 30 cycles.

LLZO‐based SSLB fabrication by co‐sintering is mainly done in two ways with LCO/LLZTO as the composite cathode: one is by field‐assisted sintering technology (FAST) and the other is free sintering in air [8, 10, 11, 12, 18]. LLZO‐based SSLBs fabricated by FAST were able to deliver high specific areal capacities, as high as 4 mAh cm^−2^ for the initial specific areal capacity, but are usually accompanied by fast capacity degradation to less than 50% within 10 cycles [11, 12, 18, 19]. By using free sintering in air, Hou et al. show that the LLZO‐based SSLB was able to achieve 80.8% capacity retention after 100 cycles with an initial specific areal capacity of 0.63 mAh cm^−2^ (6 mg LCO loading) [8]. The results suggest that the cycling performance may be further stabilized by optimizing the microstructure of the composite cathode. Nevertheless, the capacity degradation is still rather high when compared to nowadays LIBS, which needs to be further understood to better understand its degradation mechanism for better microstructure engineering.

Recent understanding of the LLZO‐based SSLB degradation mechanism is attributed to Co diffusion from LCO into LLZTO structure during the high‐temperature sintering process, which leads to high interfacial resistance to degrade the SSLB cycle performance [11, 18, 20]. Scheld et al. observed that the LLZTO separator in LLZO‐based SSLB turned bluish when prepared by FAST and brownish when prepared by rapid thermal processing [20]. The study suggests that Co substitution into LLZTO structure causes the coloration and brings detrimental effects to the used LLZTO separator, such as a decrease in ionic conductivity and lower critical current densities for dendrite formation.

For a detailed study of the Co diffusion into LLZO at high temperatures, Din et al. used Ga‐substituted LLZO single crystals and embedded them in an LCO‐powder bed to mimic the high‐temperature sintering condition [21]. They reported that Co^2+^ and/or Co^3+^ ions incorporate into the tetrahedral Li‐positions in LLZO depending on temperature and oxygen fugacity, which leads to different coloration of LLZO. By a combination of single‐crystal XRD and ICP‐OES, they found that, when a critical Co concentration is exceeded (∼0.16 per formula unit), a phase transition from cubic to tetragonal LLZO is triggered by causing Ga dissolution from the lattice due to high residual stress from lattice distortion. The release of high stress by fracture further causes Co segregation to the fracture surface, and LLZO undergoes a phase change from cubic to tetragonal phase, which has three orders of magnitude lower ionic conductivity.

A detailed investigation of experimental sections of the above‐mentioned LLZO‐based SSLB publications identified a couple of discrepancies, which make the results difficult for comparison. For example, SSLBs prepared in an air atmosphere show much higher cycle stabilities, while their TEM/EDS mappings show a clean interface between LCO and LLZTO, where Co diffusion into LLZO is hardly visible [8, 9]. When SSLB is prepared by FAST, it needs to be processed either under vacuum or an inert atmosphere to avoid graphite die oxidation. The results show bluish coloration deep into the LLZTO separator. Notably, SSLBs fabricated by FAST always require another calcination step in air at 900°C or 1050^°^C before they can be assembled into battery cells for electrochemical testing [11, 12, 18]. Thus, it is suspected that different atmospheres can cause different levels of Co‐substitution into the LLZO structure due to the different ionic radii of Co^2+^ and Co^3+^ and coordination preferences, Co^2+^ tetrahedral and Co^3+^ octahedral coordination, which also leads to different electrochemical properties of Co‐substituted LLZTO.

Here, a systematic introduction of various concentrations of Co into LLZTO was studied experimentally by sintering the materials in Ar and air atmospheres, respectively. The samples obtained were characterized based on their chemical, electrochemical, crystal structure, and microstructural properties to understand how the fabrication atmosphere affects the electrochemical performance of Co‐substituted LLZTO. Experimentally, we observed that Co is much more soluble in LLZTO when sintered in Ar than in air. Complementary density functional calculations were performed to understand the thermodynamical driving force for Co substitution at different atomic sites. Because of the computational cost, the calculations were performed for Ta‐free LLZO. Electrochemical impedance spectroscopy measurements reveal that the increased Co content in Ar‐sintered LLZTO is associated with lower total conductivity and DC electronic conductivity, which may explain why the FAST‐Fabricated SSLB always needs to be additionally calcined in air at high temperatures before electrochemical cycling can be conducted.

## 2 Results and Discussion

To determine the optimal sintering temperature for the Co_x_:LLZTO samples, where x = 0, 0.005, 0.01, 0.03, 0.05, and 0.1, the sample with the highest Co concentration, Co_0.1_:LLZTO, was sintered in air at temperatures of 970°C, 1000°C, 1050°C, 1100°C, and 1175°C. Powder X‐ray Diffraction (XRD) was used to monitor the formation of secondary phases. Figure 1a shows the XRD refinement results from Co_0.1_:LLZTO sintered at different temperatures in an air atmosphere, with a zoom‐in of the diffraction patterns between 42 and 50 degrees for better observation of secondary phase diffraction peaks. The patterns were refined by using the cubic garnet structure with space group *Ia‐3d*. The patterns show distinct diffraction peaks corresponding to LiCoO_2_ secondary phase for the samples sintered at 970°C, 1000°C, 1050°C, and 1100°C and corresponding to La_2_Li_0.5_Co_0.5_O_4_ for the sample sintered at 1175°C. Figure 1b shows the refined wt.% of LiCoO_2_ and La_2_Li_0.5_Co_0.5_O_4_ secondary phases at different sintering temperatures. The figure reveals that when the sintering temperature is increased to 1175°C, La_2_Li_0.5_Co_0.5_O_4_ was identified as the major secondary phase while the concentration of LiCoO_2_ was significantly reduced. The formation of LiCoO_2_ and La_2_Li_0.5_Co_0.5_O_4_ secondary phases indicates that the solubility of Co in garnet‐structured LLZTO is very low when the samples are sintered in air. The formation of a lanthanum (La)‐rich secondary phase, such as La_2_Li_0.5_Co_0.5_O_4_ at higher Co‐substitution levels and high sintering temperature, is consistent with previous reports identifying a reaction between LCO and LLZTO, in which LaCoO_3_ has been identified as a reaction product by differential scanning calorimetry (DSC) at ∼1085°C in air [22]. La_2_Li_0.5_Co_0.5_O_4_ has similarly been reported as a reaction product in the same system [20, 23].

**FIGURE 1:**
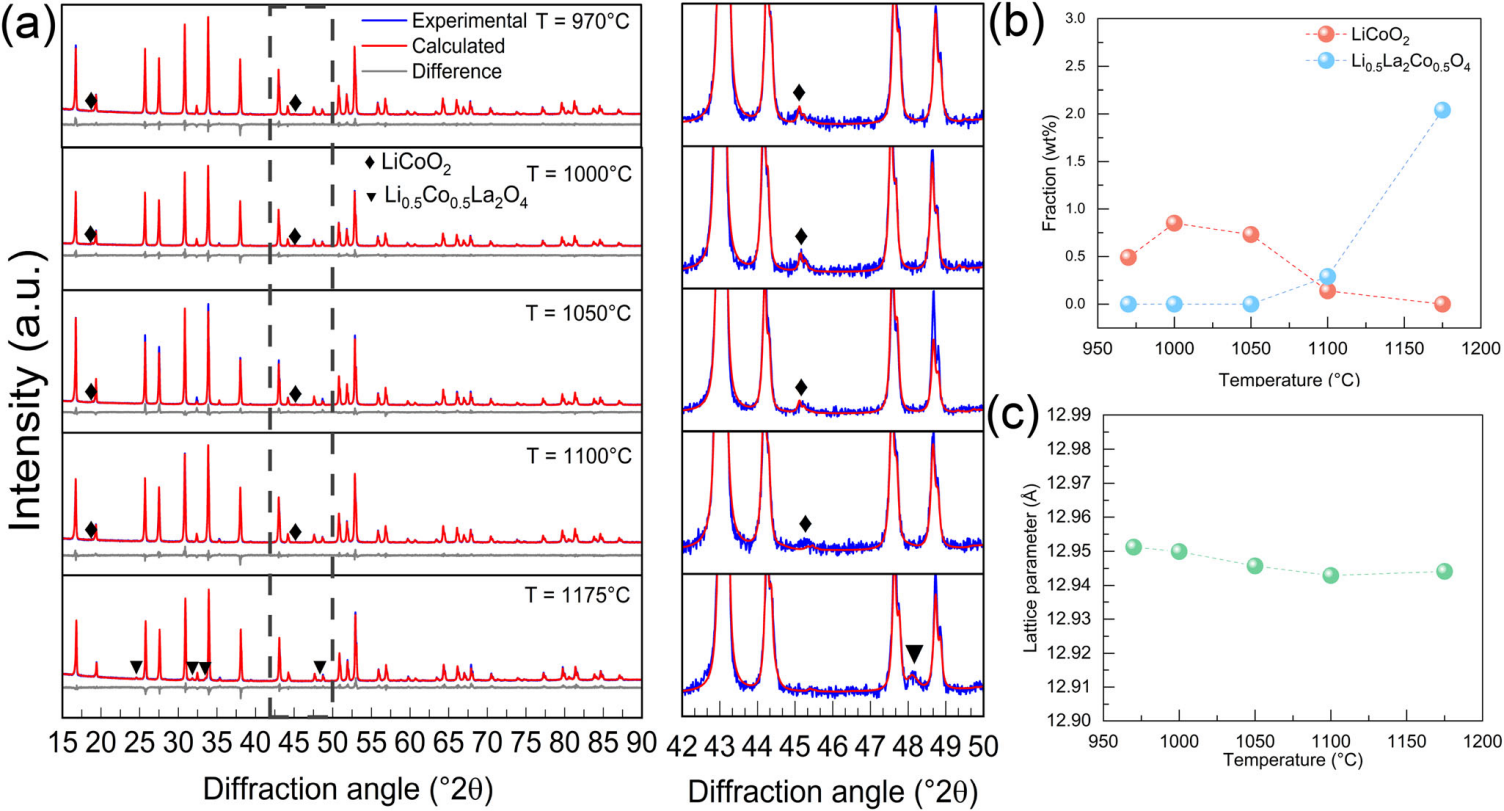
(a) X‐ray diffraction (XRD) patterns of Co_0.1_:LLZTO samples sintered in different temperatures in air (b) Weight percentage of LiCoO_2_ and Li_0.5_La_2_Co_0.5_O_4_ secondary phases in air‐sintered samples as a function of sintering temperatures, based on Rietveld refinement (c) Lattice parameters refined using the cubic *Ia‐3d* space group for the air‐sintered samples at different temperatures.

Figure 1c shows the refined lattice constants of samples sintered at different temperatures, revealing minimal variation across all sintering conditions. Based on these results, a sintering temperature of 970°C was selected for all Co*_x_*:LLZTO samples, as this minimizes the formation of La_2_Li_0.5_Co_0.5_O_4_ secondary phase. This temperature also aligns with the optimized sintering conditions for LLZTO‐based solid‐state Li battery fabrication, as established in our previous work [8, 24].

To understand the discrepant results from SSLBs fabricated under air and Ar/vacuum environments, two sets of Co_x_:LLZTO were prepared for this study, as shown in Figure 2a. Figure 2b shows the digital images of Co_x_:LLZTO pellets sintered in air and Ar atmosphere at 970°C with 10 h of dwell time, as well as their relative densities, which were measured using the Archimedes method. Both Ar‐sintered and air‐sintered samples exhibit relative densities between 85% and 90%. The most obvious difference between the two batches of samples is their color appearance, for which the samples sintered in air are khaki in color, and the samples sintered in Ar show a bluish color. The color difference can be explained by incorporating different valence states of Co ions into the garnet structure due to different oxygen partial pressures in air and Ar atmosphere, where Co^2+^ gives a blue color as that of cobalt blue CoAl_2_O_4,_ and Co^3+^ displays khaki or green coloration as that of Co_2_AlO_4_ [25]. The pellets were observed to darken progressively with increasing Co content.

**FIGURE 2:**
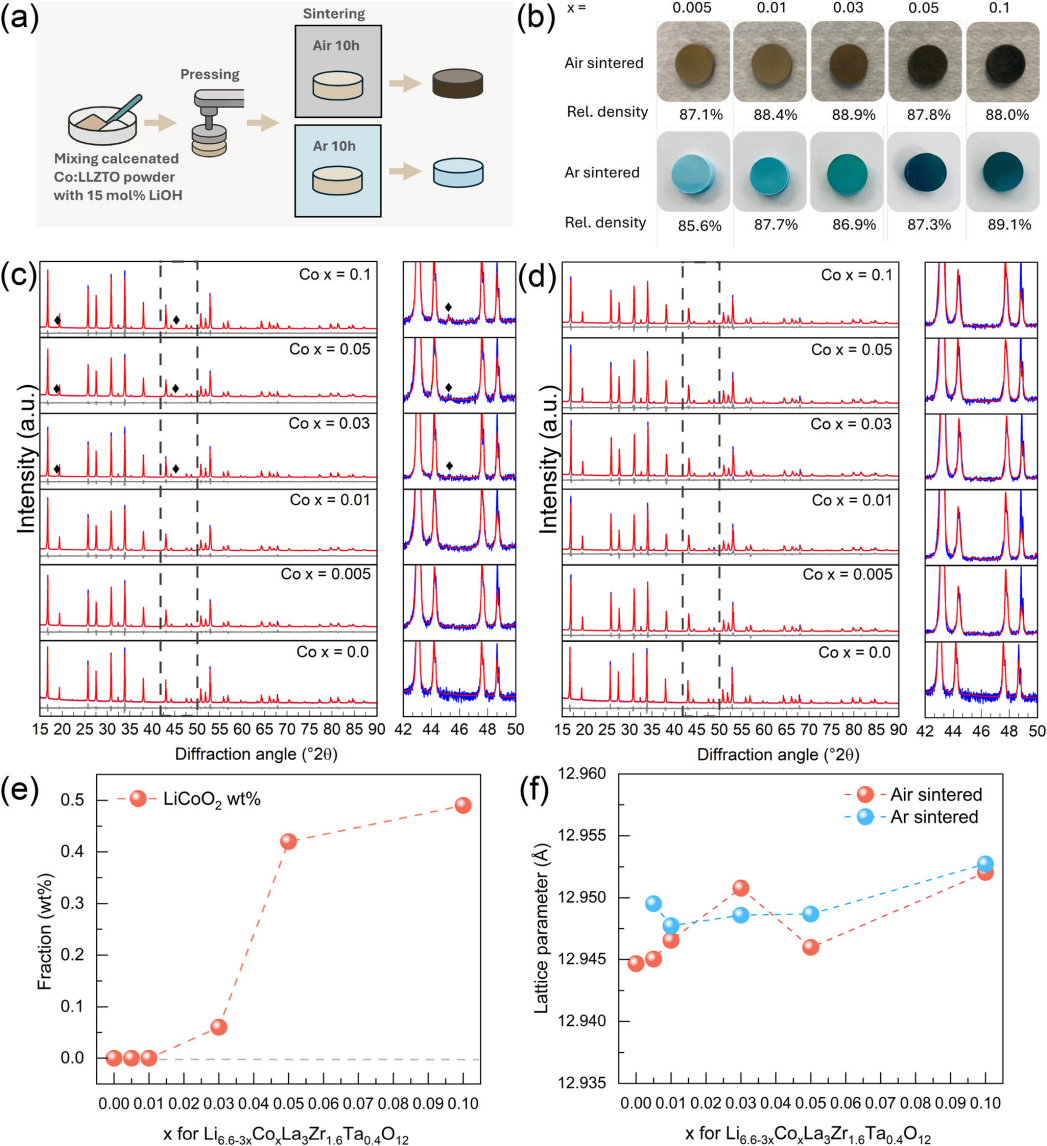
(a) Schematic illustration of the sintering procedures used to prepare air‐ and Ar‐sintered Co_x_:LLZTO samples. (b) Images of Co_x_:LLZTO pellets after sintering in air (khaki) and Ar (blue) atmospheres across varying Co concentrations in addition to the relative density measured using the Archimedes method. (c) XRD patterns of Co_x_:LLZTO sintered in air, (d) XRD patterns of Co_x_:LLZTO sintered in Ar. For both a zoom‐in of the range 42° ≤ 2Θ ≤ 50° is shown. (e) Weight fraction (wt.%) of the LiCoO_2_ secondary phase as a function of Co concentration obtained from Rietveld refinement. (f) Refined lattice parameter a as a function of Co concentration.

Figure 2c,d shows the XRD refinement results for the Co_x_:LLZTO samples sintered in air and Ar, respectively, with a zoom on the diffraction patterns between 42 and 50 degrees for better observation of the diffraction peaks of the secondary phases. The patterns were primarily refined using the cubic garnet structure with the *Ia‐3d* space group. For Co_x_:LLZTO samples sintered in the air atmosphere, the LCO phase was detected as the secondary phase when the Co concentration was greater than x = 0.01. Figure 2e shows the wt.% fraction of the LCO secondary phase as refined from Rietveld. This indicates that the solubility limitation of Co in LLZTO should be between 0.01 and 0.03 Co per formula unit Co_x_:LLZTO when sintering is performed in air. In contrast, no secondary phase peaks were observed in the Ar‐sintered Co_x_:LLZTO, suggesting higher solubility of Co (Figure 2e). This is probably because Co^2+^ has an ionic radius similar to that of Li^+^ (Shannon radii for sixfold coordination: Co^2+^ high spin 74.5 pm, Li^+^ 76 pm), whereas Co^3+^ is considerably smaller (54.5 pm low spin, 61 pm high spin), which facilitates the substitution of Co^2+^ on Li sites [26]. To elucidate the thermodynamic preference for Co incorporation into the LLZO lattice, density functional theory (DFT) calculations were performed for Ta‐free cubic LLZO (see Experimental Section) by substituting Co at different atomic sites, i.e. Li (tetrahedral, octahedral, and distorted non‐coplanar), Zr, and La sites. The calculations show that Co^2^^+^ and Co^3^^+^ preferentially occupy the Li and Zr sites (see Table 1), with a local strongly distorted triangular non‐coplanar and octahedral Co–O coordination environment, respectively. These site‐specific occupancy preferences also give rise to distinct electronic characteristics, as discussed in the following sections. The valence of Co and its position determine the charge‐balancing defects and, consequently, the stoichiometry of Co‐substituted LLZTO which is listed in Table S1. A Co^2+^ ion at a Li site carries a net positive charge, which is balanced by an additional Li vacancy (Li_6.6−2x_Co_x_La_3_Zr_1.6_Ta_0.4_O_12_). A Co^3+^ ion at a Li site is compensated by two Li vacancies (Li_6.6−3x_Co_x_La_3_Zr_1.6_Ta_0.4_O_12_), which is the nominal composition used for synthesis. A Co^3+^ ion at the Zr site is compensated by a Li interstitial at vacant sites on the Li sublattice (or, alternatively, by one oxygen vacancy per two Co^3+^ ions). A Co^3+^ ion at a La site requires no compensation. Oxygen vacancies cannot compensate for Co^2+^ at Li sites as they have the same charge. For the experimental compositions, Table S1 assumes complete Co incorporation into LLZTO.

**TABLE 1:** DFT‐predicted formation energies and lattice parameters for Co substitution at different cation sites in LLZO. As described in the Experimental section, a reduced primitive cell was employed to compare the relative formation energies of Co substitution. Charge neutrality was maintained by adjusting the Li stoichiometry through the formation of either Li interstitials or Li vacancies in the vicinity of the Co dopant, depending on the substitution site. Multiple local atomic configurations were evaluated to identify the lowest‐energy structure. The corresponding Co defect formation energies and relaxed lattice parameters for Co occupying the different cation sites are summarized here.

Co occupancy	Formation energy (eV/Co atom)	Lattice Parameters						Volume (Å^3^)
		a (Å)	b (Å)	c (Å)	α (°)	β (°)	γ (°)	
Co^2+^ at Li site	−0.92	11.281	11.291	11.295	110.16	108.96	109.42	1105.70
Co^3+^ at Li site	−0.44	11.262	11.289	11.265	109.89	109.03	109.48	1102.76
Co^3+^ at La site	−0.48	11.297	11.355	11.269	109.51	108.04	111.06	1109.51
Co^3+^ at Zr site	−1.31	11.275	11.283	11.286	110.84	107.89	109.53	1106.64
Pristine LLZO	N.A.	11.267	11.276	11.289	109.93	108.54	109.92	1104.29

Figure 2f displays the refined lattice parameters for both air‐ and Ar‐sintered samples, showing minimal variation with increasing Co compared to LLZTO (x = 0). The lack of a significant change in lattice parameter, particularly in the Ar‐sintered samples, is consistent with the substitution of Co on Li sites: according to the empirical formula of Strocka et al. [27], Co on the Li site (Co^2+^ or Co^3+^) is not expected to change the lattice parameter noticeably, whereas Co^3+^ on the Zr or La site would decrease it slightly (Figure S1). The DFT calculations likewise predict only small volume changes, about 0.2% for Co^3+^ at the Zr site and 0.13% for Co^2+^ at the Li site (Table 1). Since the Co contents reached experimentally are even lower than that of the DFT cell, these changes are below the resolution of laboratory XRD, so that the lattice parameter alone cannot establish the site or the valence of Co.

To investigate the effect of Co substitution on the microstructure of Co_x_:LLZTO, scanning electron microscopy (SEM), Electron Backscatter Diffraction (EBSD), energy‐dispersive X‐ray spectroscopy (EDS), and Raman spectroscopy were performed. Figure 3a,d shows the SEM images and Co EDS maps of air‐ and Ar‐sintered Co_x_:LLZTO for x = 0.005, 0.03, and 0.1 at 970°C. While EBSD (Figure S2) reveals no change in grain size distribution with sintering atmosphere and Co‐substitution (∼5 µm), the SEM images (Figure 3a,d) reveal distinct pore morphologies between the two sintering atmospheres. Air‐sintered samples display large pores, whereas Ar‐sintered samples contain a greater number of smaller, more distributed pores, as confirmed by the mean pore area data in Figure S3. Ar‐sintered samples have mean pore area of approximately 30 µm^2^ while that for air‐sintered samples the mean pore area of approximately 70 µm^2^. The microscopical porosity is determined to be around 10% for air‐sintered samples and 15% for Ar‐sintered samples, which corresponds quite well with the relative densities measured using the Archimedes method (Table 3).

**FIGURE 3:**
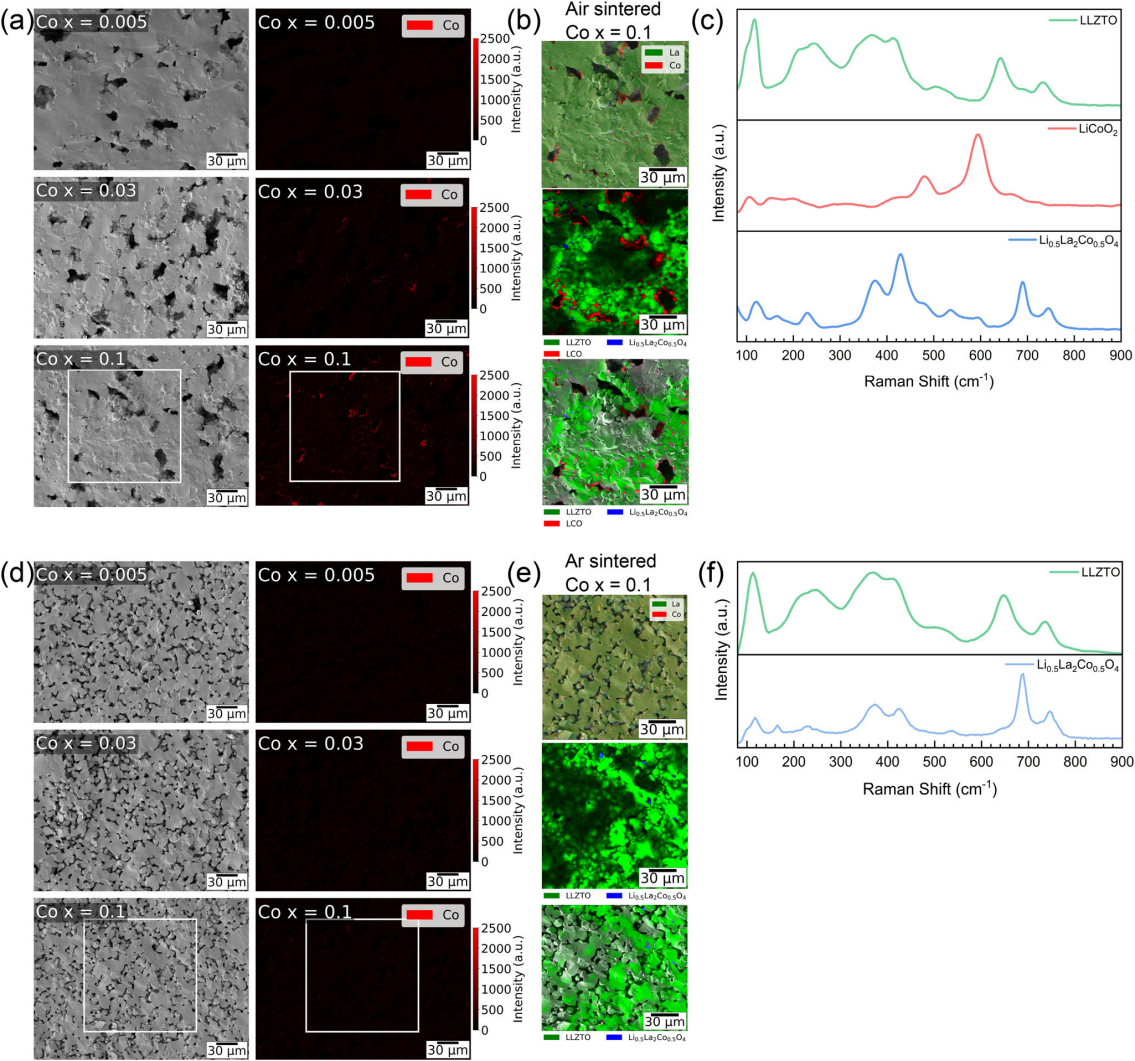
(a, d) SEM images and EDS elemental maps of samples with varying Co concentrations sintered in air (a) and Ar (d). (b, e) EDS and Raman maps of the selected regions in air‐sintered (b) and Ar‐sintered (e). The selected regions are marked in white squares in EDS maps of Co_0.1_:LLZTO samples in (a) and (d). (c, f) Corresponding Raman spectra showing cubic LLZTO with LCO and La–Co‐rich phases in air‐sintered samples (c), and cubic LLZTO with La–Co‐rich phases in Ar‐sintered samples (f).

SEM‐EDS mapping (Figure 3a) revealed Co segregation along grain boundaries and pore surfaces for x = 0.03 and above. This is consistent with the detection of LCO as a secondary phase by XRD at x ≥ 0.03. For the Ar‐sintered sample at x = 0.1, only a few Co‐rich spots appear on the Co EDS map. Figure 3b presents the superposition of SEM/EDS maps (top), Raman map (middle), and superposition of SEM/Raman maps (bottom) of air‐sintered Co_0.1_:LLZTO as marked by the white square box in Figure 3a. Three distinct phases: cubic LLZTO, LCO, and Li_0.5_La_2_Co_0.5_O_4_ were identified in air‐sintered Co_0.1_:LLZTO by their detected average Raman spectra as shown in Figure 3c. The cubic LLZTO phase is confirmed by characteristic peaks at 115 cm^−^^1^ corresponding to La sublattice vibration, 213, 242, 371, and 410 cm^−^^1^ corresponding to internal Li─O modes, 649 cm^−^^1^ corresponding to Zr─O stretching, and 737 cm^−^^1^ corresponding to Ta─O stretching [28, 29, 30, 31]. The LCO phase is evidenced by peaks at 484 cm^−^^1^ corresponding to the O─Co─O bending mode, 595 cm^−^^1^ corresponding to the Co─O stretching mode, and 658 cm^−^^1^ corresponding to the Co─O stretching mode characteristic of possible impurity of Co_3_O_4_ [32, 33]. The Li_0.5_La_2_Co_0.5_O_4_ phase is characterized by peaks at 385, 431, and 690 cm^−^^1^ [20]. It is worth mentioning that a laser wavelength of 785 nm was used for excitation to avoid the photoluminescence effect from Co substituting into the garnet structure for the Raman mapping [20, 34]. This allows us to separate the contribution from Li_0.5_La_2_Co_0.5_O_4_ from LLZTO by using the peak position at 690 cm^−1^ (Figures 3 and 5). The Raman mapping shows that LCO is the major secondary phase, while Li_0.5_La_2_Co_0.5_O_4_ is the minor secondary phase in only a few areas in the air‐sintered Co_0.1_:LLZTO. When overlapping the SEM, EDS, and Raman mapping together, it can be clearly seen that the Co‐rich area in EDS is the same as where LCO was identified by Raman mapping, mainly along grain boundaries and pore surfaces, Figure 3b.

Similar correlative SEM/EDS/Raman‐mappings for the Ar‐sintered Co_x_:LLZTO are shown in Figure 3d,e. Only two phases were identified: cubic LLZTO and Li_0.5_La_2_Co_0.5_O_4_ secondary phase, which only appears in a few spots for x = 0.1, indicating a higher solubility of Co in the LLZTO that agrees with XRD results, showing no secondary phases (Figure 2d). Their corresponding Raman spectra extracted from the Raman mapping are presented in Figure 3f. The Raman mapping also discloses that the Co‐rich area in the EDS mapping of the Ar sintered sample is where the Li_0.5_La_2_Co_0.5_O_4_ phase was identified.

To compare the solubility of Co in LLZTO under different sintering atmospheres, EDS‐point measurements within grain centers were conducted on the Co_0.1_:LLZTO samples. Representative measurement positions are shown in Figure 4a,b. The corresponding quantification tables in Figure 4c,d show that the Co atomic percentage (at%) in the Ar‐sintered sample (0.47 at.%) appears higher than that in the air‐sintered sample (0.11 at.%), suggesting that Co is more soluble in LLZTO in an Ar‐sintering atmosphere than in an air‐ sintering atmosphere. The overlaid EDS spectra in Figure 4e support the quantification results, revealing a distinct difference in Co Kα intensities at around 6.9 keV between the two sintering conditions. Figure 4f shows the kernel density estimates (KDEs), which statistically extend this analysis across all 26 measurement points. As can be seen from the figure, the Ar‐sintered sample exhibits an overall higher atomic percentage of Co ∼0.44 at% on average, which corresponds to x ∼ 0.08, compared to the air‐sintered sample ∼0.1 at% on average, which corresponds to x ∼ 0.02, which corresponds to the solubility limit estimated from XRD. This demonstrates that incorporating Co into LLZTO under reducing conditions is far more effective than under oxidizing conditions. The figure also shows the distribution of Co at%. values in the Ar‐sintered sample are relatively broad, suggesting a degree of inhomogeneity in Co incorporation into LLZTO.

**FIGURE 4:**
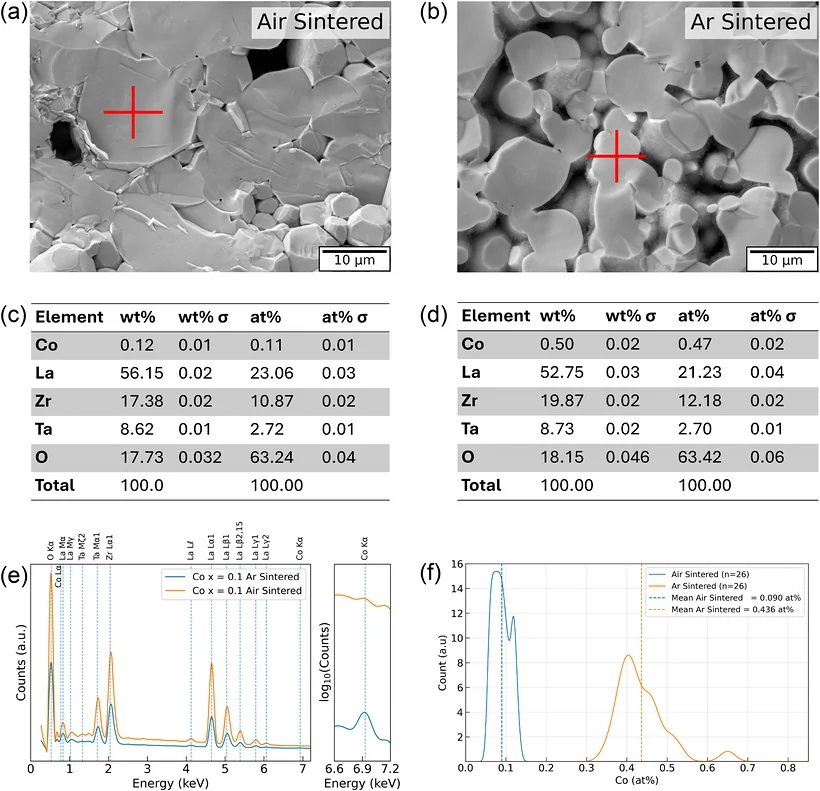
EDS‐analysis of Co_0.1_:LLZTO samples sintered in air and Ar. Representative measurement positions on (a) air‐ and (b) Ar‐sintered samples with corresponding quantification tables in (c) and (d). (e) Overlaid ED‐spectra from the two measurement points. (f) Kernel density estimates (KDEs) of elemental composition across 26 measurement points per sample.

The Co content measured by EDS at the grain centers of all compositions is summarized in Table 2 (ten grains per sample). For x ≤ 0.01, the expected Co content for complete incorporation (≤ 0.06 at%, Table S1b) is below the detection limit of the spot analysis, and no Co was detected in either atmosphere. For x ≥ 0.03, the two atmospheres differ significantly: in the Ar‐sintered samples, the Co content of the grains increases with x from 0.12 ± 0.02 at% (x = 0.03) to 0.42 ± 0.04 at% (x = 0.1), whereas in the air‐sintered samples it does not exceed 0.1 at% for x = 0.03, 0.05, and 0.1, i.e., it saturates at around 0.02 Co per formula unit. This value is in line with the solubility limit of 0.01 < x < 0.03 derived from the appearance of LCO in the diffraction patterns (Figure 2e). When more Co is added, LCO and Li_0.5_La_2_Co_0.5_O_4_ are formed and appear at grain boundaries and pore surfaces (Figure 3a–c). Under reducing conditions, the incorporation of Co into the LLZTO lattice is thus 2.5 to 5 times higher than under oxidizing conditions.

**TABLE 2:** Co content inside the LLZTO grains, as determined by SEM‐EDS spot analysis (15 kV, ten grains per sample, mean ± standard deviation).

Nominal x	Air: Co in grains (at%)	Ar: Co in grains (at%)
0.005	Not detected	Not detected
0.01	Not detected	Not detected
0.03	0.05 ± 0.03	0.12 ± 0.02
0.05	0.10 ± 0.03	0.32 ± 0.06
0.1	0.09 ± 0.02	0.42 ± 0.04

Figure 5a,b shows the Raman spectra of Co_x_:LLZTO with varying Co concentrations sintered in air and Ar by using a 532 nm excitation wavelength. All samples display characteristic peaks associated with the cubic garnet phase. In air‐sintered samples, a peak at 693 cm^−^^1^ starts to be observable when Co concentration is x = 0.03, which is associated with the photoluminescence from Co incorporation into the LLZTO lattice, as this peak dramatically diminished when switching the excitation wavelength from 532 nm to 785 nm in Figure 5c [10]. In contrast, the peak at 693 cm^−^^1^ is not observed from the Ar‐sintered Co_x_:LLZTO, even at Co concentration x = 0.1, Figure 5b. This feature therefore occurs only in samples that were sintered under oxidizing conditions. Since the solubility limit of Co in air is low (Table 2), it must originate from the small amount of Co that is incorporated into the lattice under these conditions.

**FIGURE 5:**
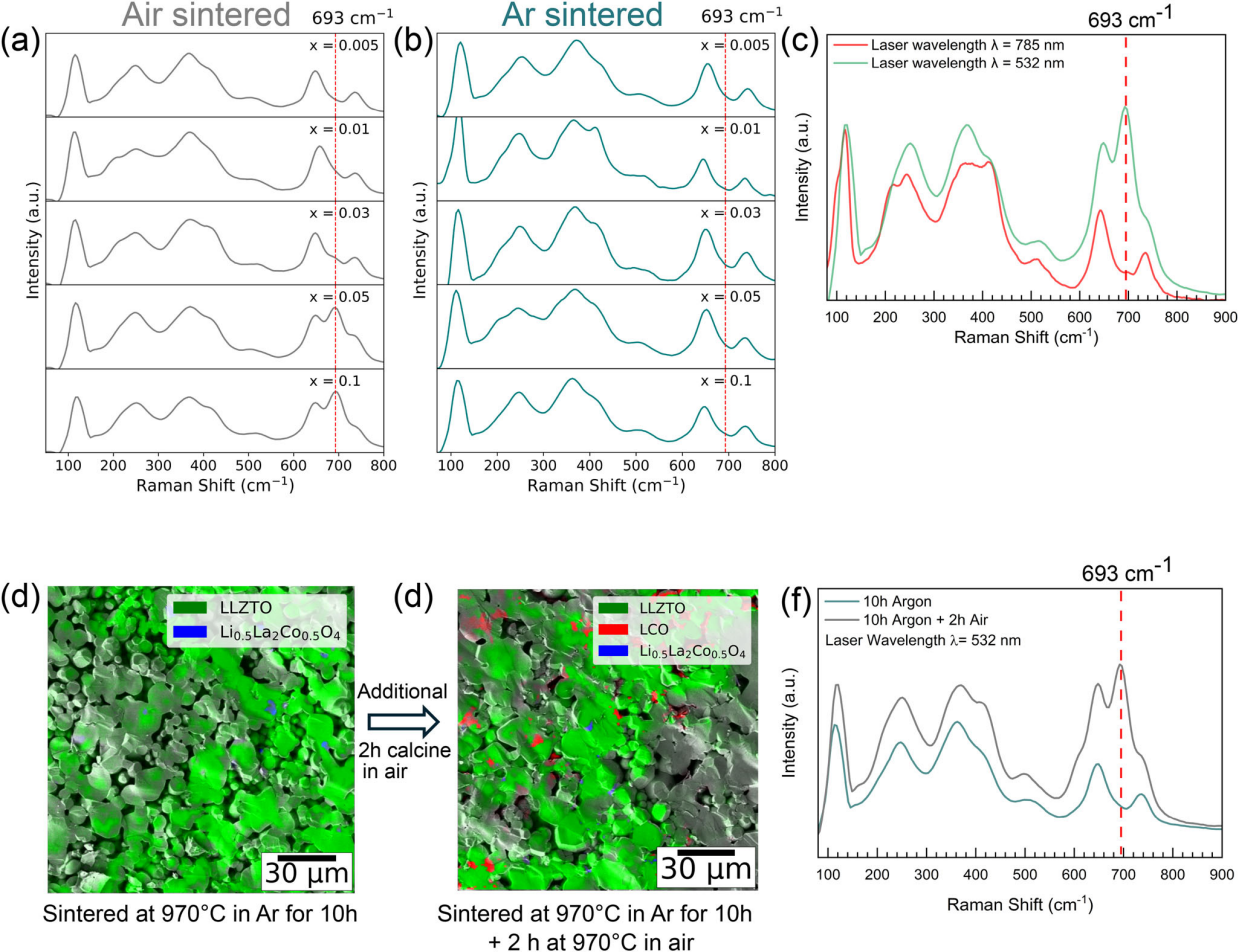
(a) and (b) Co_x_:LLZTO Raman spectra of samples sintered in air and Ar atmospheres respectively. (c) Raman spectra of air‐sintered Co_0.1_:LLZTO acquired using 532 nm and 785 nm excitation wavelengths. (d) and (e) show Raman maps of the Ar‐sintered Co_0.1_:LLZTO, before and after an additional 2 h of sintering in air, respectively. (f) Corresponding Raman spectra extracted from the mapped regions shown in (d) and (e).

To verify this hypothesis, a Co_0.1_:LLZTO was first sintered at 970°C in an Ar atmosphere with 10 h dwell time for Raman measurement (Figure 5d). The Raman mapping detected only a few Li_0.5_La_2_Co_0.5_O_4_ spots without any LCO, the same as that in Figure 3e. Figure 5f shows the corresponding Raman spectrum extracted from the mapped LLZTO regions obtained using a 532 nm excitation wavelength. The spectrum shows no photoluminescence feature at 693 cm^−1^. The same sample was subjected to an additional 2‐h heat treatment in air at 970°C. After the heat treatment, the Raman mapping of the sample revealed the formation of LCO and minor Li_0.5_La_2_Co_0.5_O_4_ secondary phases (Figure 5e), which is evidence of Co segregated out of the LLZTO structure, the same chemical features as those in Figure 3b. As shown in Figure 5f, the corresponding Raman spectrum extracted from the mapped LLZTO regions after the 2‐h heat treatment shows that the 693 cm^−^^1^ peak clearly emerges, which indicates that part of the Co remains in the LLZTO lattice under these oxidizing conditions.

The appearance of the peak at 693 cm^−^^1^ exclusively under oxidizing conditions contradicts the findings of Scheld et al., who attribute this signal to Co ions in a mixed or partially reduced Co oxidation state within the LLZO lattice [20]. They concluded from the observation that subsequent air annealing, which fully oxidizes Co to Co^3+^, causes the signal to almost completely disappear. They, therefore, conclude that the photoluminescence signal is specifically caused by the partially reduced Co^x+^ state. However, this work suggests the opposite conclusion, as the signal appears under air‐sintering conditions but is absent under Ar sintering conditions.

To understand the impact of Co substitution on the ionic and electronic conductivities of Co_x_:LLZTO, electrochemical impedance spectroscopy (EIS) and chronoamperometric measurements were applied to Co_x_:LLZTO samples, which were sintered at 970^°^C. Figure 6a shows the Nyquist plots of air‐ and Ar‐ sintered Co_0.1_:LLZTO and LLZTO measured at −20°C, along with the fitting results for Ar‐sintered Co_0.1_:LLZTO, while the fits of air‐sintered Co_0.1_:LLZTO and LLZTO and Ar‐sintered LLZTO can be found in Figure S4, respectively. The contributions from bulk and grain boundaries can be clearly separated for Ar‐sintered Co_0.1_:LLZTO and LLZTO, where C_p_ ∼ 10^−11^ F cm^−1^ is assigned for bulk contribution and where C_p_ ∼ 10^−8^ F cm^−1^ is assigned for grain boundary contribution [35]. However, the assignment of the contribution from fitting capacitance values for air‐sintered Co_0.1_:LLZTO encounters difficulty because of the similar fitting capacitance values at high‐ and mid‐frequency ranges, which could be relative to the formation of LCO secondary phase in the sample, Figure S4c [35]. Nevertheless, the tails for individual Nyquist plots were confirmed to be the contribution of electrode polarization (blocking) by their fitting capacitance in the range between 10^−7^ and 10^−6^ F cm^−1^ [35]. Therefore, only the temperature‐dependent total conductivities of Co_x_:LLZTO were derived from the EIS measurements and are plotted in Figure 6b,c.

**FIGURE 6:**
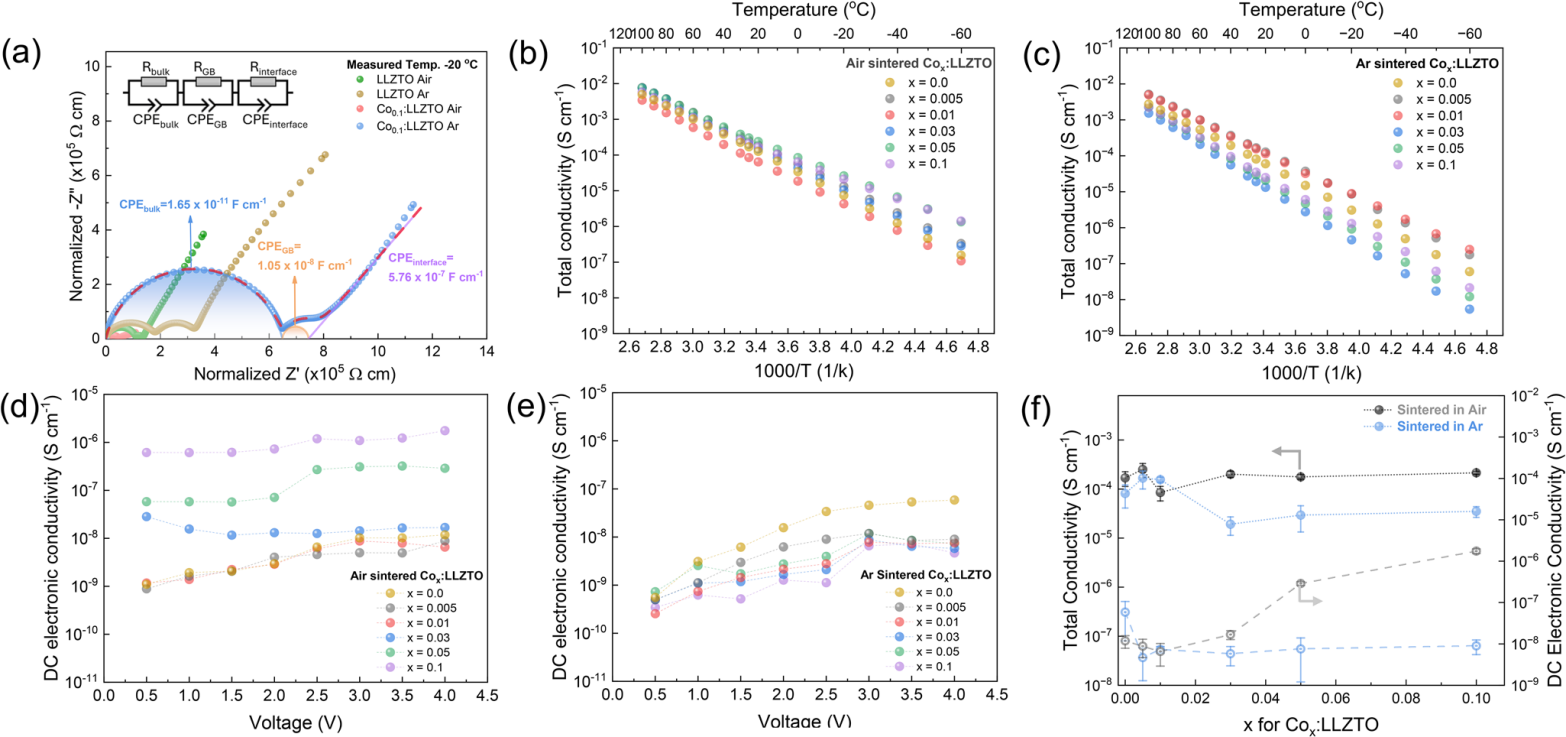
(a) Nyquist plots of air‐ and Ar‐sintered LLZTO and Co_0.1_:LLZTO. (b) Temperature‐dependent total conductivity of Co_x_:LLZTO samples sintered in air and (c) in Ar. (d) DC electronic conductivity of air‐sintered Co_x_:LLZTO samples and (e) Ar‐sintered Co_x_:LLZTO samples measured as a function of applied voltage at 25°C. (f) Total conductivity (closed circles) and DC electronic conductivity (open circles) by applying 4 V bias at 25°C as a function of Co concentration for air‐ and Ar‐sintered Co_x_:LLZTO samples.

The DC electronic conductivities of Co_x_:LLZTO were measured by chronoamperometry at 25°C (Figure 6d,e). For the Co_x_:LLZTO sintered in Ar, the electronic conductivities are about 5 × 10^−10^ S cm^−1^ at a bias of 0.5 V and increase slowly to about 5 × 10^−9^ S cm^−1^ at 4.0 V, except for the Ar‐sintered LLZTO, which reaches ∼5 × 10^−8^ S cm^−1^ (Figure 6e). The air‐sintered LLZTO and Co_x_:LLZTO with x ≤ 0.03 show 1–5 × 10^−9^ S cm^−1^ at 0.5 V and increase similarly to ∼10^−8^ S cm^−1^ at 4 V (Figure 6d). The increase with bias reflects the change of the Li chemical potential at the ion‐blocking electrodes, which changes the concentration of the electronic charge carriers, and is in agreement with previous reports [36, 37, 38]. For the air‐sintered samples with x ≥ 0.05, the electronic conductivity is one to two orders of magnitude higher already at low bias and reaches ∼10^−7^ S cm^−1^ for x = 0.05 and ∼10^−6^ S cm^−1^ for x = 0.1 at 4 V (Figure 6d); for x = 0.1 it varies by less than a factor of two with the bias. Although DFT calculations predict the general formation of in‐gap electronic states arising from hybridization between Co 3d and O 2p orbitals (Figure S5), the resulting increase in the intrinsic electronic conductivity of LLZO is expected to remain modest. Therefore, the observed orders‐of‐magnitude increase in the electronic conductivity with increasing Co content is more likely attributable to the formation of the LCO secondary phase, which has an electronic conductivity of about 1 × 10^−3^ S cm^−1^ at room temperature [39]. Figure S8 shows the correlation of the electronic conductivity with the LCO content from the Rietveld refinement.

In general, the total conductivities of air‐sintered Co_x_:LLZTO remain similar at ∼ 1 × 10^−4^ S cm^−1^ at 25°C, Figure 6F. This can be explained by the low Co solubility in air‐sintered Co_x_:LLZTO (x ≈ 0.02 from EDS, Figure 4 and Table 2). The LCO at the grain boundaries apparently does not impede the Li‐ion transport noticeably, since the total conductivity does not decrease with increasing LCO content (Figure 6f). When the substitution of Co into LLZTO structure increases to x ≥ 0.03 under Ar‐sintering atmosphere, their total conductivity decreases to ∼2 × 10^−5^ S cm^−1^ at 25°C, which agrees with Din et al. that blue color LLZO gives lower conductivity than the orange/khaki one [21]. We attribute this to the occupation of Li sites by Co, which reduces the Li mobility.

Figure S9 shows the Co content of the grains together with the total and DC electronic conductivities. In the Ar‐sintered samples, the drop in total conductivity occurs at x = 0.03, where Co is first detected in the grains; for x ≤ 0.01 the expected Co content (≤ 0.06 at%, Table S1b) is below the detection limit of EDS. The total conductivity does not decrease further as the Co content of the grains rises from 0.12 to 0.42 at% (Figure S9a). The reduction in total conductivity in the Ar‐sintered samples is likely caused by Co occupation of Li^+^ sites, which disrupts Li‐ion transport pathways and increases the activation energy (Table 3). Since even 0.42 at% Co corresponds to the replacement of only about 1% of the Li ions, the effect cannot be a simple blocking of Li sites; it presumably involves defect interactions on the Li sublattice, e.g., the association of Co^2+^ with the charge‐compensating Li vacancies, which saturate at low Co contents. This remains to be clarified. The DC electronic conductivity of the Ar‐sintered Co_x_:LLZTO samples changes very little with the Co content (4.7–9.0 × 10^−9^ S cm^−1^ at 4 V for x = 0.005–0.1). In contrast, a larger amount of LCO at the grain boundaries of air‐sintered samples increases their DC electronic conductivity by one to two orders of magnitude for x ≥ 0.05, while the Co content of their grains remains at approximately 0.1 at%. Therefore, the electronic conductivity does not follow the Co content of the LLZTO lattice. Instead, it follows the amount of LCO (see Figure S8). Table 3 summarizes the total conductivity (σ_total_), DC electronic conductivity (σ_e_), activation energy, and relative density of Co_x_:LLZTO samples sintered in air and in Ar atmosphere. It is worth mentioning that the relative densities of the Co_x_:LLZTO samples sintered in air and Ar at 970°C range only from 86% to 89%, which is lower than the typically reported LLZTO for a relative density > 93% [34]. The lower total conductivity of Co_x_:LLZTO sintered at 970°C can be partly attributed to their lower relative densities, alongside the effects of Co substitution. Nevertheless, the standard deviation of the relative densities of the Co‐substituted samples is only 1.03% (0.67% within the air‐sintered and 1.27% within the Ar‐sintered series), which should serve as a fairly insightful comparison that the incorporation of Co into the LLZTO structure, as observed for the Ar‐sintered samples, dramatically decreases its total and DC electronic conductivities.

**TABLE 3:** Total conductivity (σ_total_) and DC electronic conductivity (σ_e_) at 25°C, activation energy, and relative density by Archimedes method of Co_x_:LLZTO samples as a function of Co concentration (*x*) for samples sintered in air and Ar.

Co_x_:LLZTO	Air: σ_total_ at 25°C (S cm^−^^1^)	Air: Activation Energy (eV)	Air: σ_e_ at 25°C with 4 V bias (S cm^−^^1^)	Air: Relative density (%)	Ar: σ_total_ at 25°C (S cm^−^^1^)	Ar: Activation Energy (eV)	Ar: σ_e_ at 25°C with 4 V bias (S cm^−^^1^)	Ar: Relative density (%) (Archimedes)
x = 0	1.69 × 10^−^^4^	0.47	1.18 × 10^−^^8^	87.6	8.11 × 10^−^^5^	0.49	5.87 × 10^−^^8^	86.7
x = 0.005	2.64 × 10^−^^4^	0.46	8.81 × 10^−^^9^	87.1	1.67 × 10^−^^4^	0.46	4.66 × 10^−^^9^	85.6
x = 0.01	8.64 × 10^−^^5^	0.47	6.55 × 10^−^^9^	88.4	1.55 × 10^−^^4^	0.45	7.31 × 10^−^^9^	87.7
x = 0.03	2.43 × 10^−^^4^	0.46	1.68 × 10^−^^8^	88.9	1.92 × 10^−^^5^	0.57	5.78 × 10^−^^9^	86.9
x = 0.05	3.03 × 10^−^^4^	0.43	2.88 × 10^−^^7^	87.8	2.94 × 10^−^^5^	0.55	7.56 × 10^−^^9^	87.3
x = 0.1	2.14 × 10^−^^4^	0.38	1.75 × 10^−^^6^	88.0	2.49 × 10^−^^5^	0.52	8.97 × 10^−^^9^	89.1

## 3 Conclusions

This study examines the impact of sintering atmosphere on the microstructure and electrochemical performance of Co‐substituted LLZTO. A combined analysis of XRD, EDS, and Raman spectroscopy shows that Co is more soluble in LLZTO under an Ar atmosphere than under air atmosphere. The increased solubility of Co in Ar‐sintered samples is associated with a reduction of the total conductivity by a factor of three to four and of the DC electronic conductivity by about one order of magnitude relative to the unsubstituted LLZTO. In contrast, the total conductivity of the air‐sintered samples shows no systematic change, while their DC electronic conductivity increases by up to two orders of magnitude. The increase in DC electronic conductivity of air‐sintered samples is attributed to the buildup of LCO secondary phase at grain boundaries and pore surfaces when the solubility limit of Co^3+^ is reached. The reduction in total conductivity in Ar‐sintered samples could be due to the substitution of Co^2+^ at the Li sites, which disrupts Li‐diffusion pathways. By comparing DFT formation energies for Co‐substitution at different atomic sites in pristine LLZO, it is suggested that Co^2^^+^ prefers to reside at the distorted, non‐coplanar Li site, while Co^3^^+^ prefers to occupy the octahedral Zr site. While Co^2^^+^ substitution at the Li site has a negligible impact on the electronic DOS, Co^3^^+^ substitution introduces electronic states within the bandgap, which may facilitate electronic transport. However, the corresponding increase in LLZO's intrinsic bulk DC electronic conductivity is expected to remain modest due to the relatively low Co concentration and localized nature of the defect states. The experimentally observed increase in LCO secondary phase formation in the air‐sintered samples suggests that the enhanced DC electronic conductivity is dominated by the formation of electronically more conductive LCO phases and the accompanying microstructural evolution, rather than by Co substitution within the LLZO lattice itself. Overall, our analysis shows that the sintering atmosphere is an important factor to consider since it influences the solubility of Co into LLZTO, and in turn, the electrochemical performance of LLZTO. These results suggest that a post‐calcination in air of LCO–LLZTO composite cathodes fabricated by FAST could restore part of the ionic conductivity of the LLZTO by oxidation of Co^2+^ and its segregation to the grain boundaries as LCO. This hypothesis remains to be verified by measurements before and after post‐calcination on FAST‐fabricated samples.

## 4 Experimental

### 4.1 Material Synthesis

Li_6.6‐3x_Co_x_La_3_Zr_1.6_Ta_0.4_O_12_ samples were prepared by solid‐state reaction under air. LiOH∙H_2_O (Merck, 98%), La_2_O_3_ (Merck, 99.9%, pre‐dried at 900°C for 10 h), ZrO_2_ (Treibacher, 99.5%), Ta_2_O_5_ (Inframat, 99.95%), and Co_3_O_4_ (Merck, 99.5%) were mixed in stoichiometric amounts to achieve Co concentrations of x = 0, 0.005, 0.01, 0.03, 0.05 and 0.10 in Li_6.6‐3x_Co_x_La_3_Zr_1.6_Ta_0.4_O_12_, with 15 mol% LiOH·H_2_O in excess. The prepared powder was dry‐mixed for the calcination processes. The calcination processes comprised three‐step calcinations, once at 850°C and twice at 1000°C, with a dwell time of 20 h. Grinding and pressing were repeated between the calcination steps. Then the obtained pellet was again ground and sieved through a 100 µm sieve. The obtained powder was pressed into a pellet using a die with a diameter of 12 mm and uniaxial pressing by applying 150 MPa. Before the final sintering step, the pellets were pressed by an isostatic press with 500 MPa to further increase the density. The sintering of Co_x_:LLZTO was carried out at 970°C for 10 h in air or Ar. Additional sintering temperatures of 970°C, 1000°C, 1050°C, 1100°C, and 1175°C were also investigated exclusively in air to examine the temperature‐dependent formation of secondary phases.

### 4.2 X‐Ray Powder Diffraction

The powder X‐ray diffraction (PXRD) patterns were recorded by a lab diffractometer (Empyrean, Panalytical) with a copper anode (K_α_ wavelength 1.54 Å). The measurements were carried out in a Bragg‐Brentano geometry on a silicon wafer. All samples were measured in a 2Θ range from 10° to 100° with a step size of 0.0084 degrees. The quantitative phase analyses (QPA) based on the PXRD data were conducted using the software package Diffrac.Topas Version 7 (Bruker). The lattice parameters were determined by using the Rietveld method. To determine the phases, QPA were performed for all samples, for which lattice parameters and scale factors were refined.

### 4.3 Electrochemical Analysis

Electrochemical Impedance Spectroscopy (EIS) was used for determining the total conductivity of fabricated samples by using the Turnkey Broadband System Concept 80 (Novocontrol, Germany). The EIS measurements were done using the Alpha‐A analyzer, which measured in the frequency range of 10 MHz ∼ 1 Hz, in the Broadband System Concept 80 system. Sputtered gold thin films were used as electrodes for the total conductivity measurements. The impedance spectra were collected in the temperature range from −60°C to 100°C with a step size of 10°C. A perturbation electric field of 20 mV/mm was used for the EIS measurements. The EIS data were analyzed by “ZView” (Scribner Associates, USA) using 3 series connected R/CPE circuits. The bulk, grain‐boundary, and electrode contributions were assigned according to their fitted capacitances of approximately 10^−^^1^ ^1^, 10^−^^8^, and 10^−^^7^–10^−^^6^ F cm^−^^1^, respectively [35]. For the DC electronic conductivity measurements, a BioLogic VMP‐300 multipotentiostat, combined with a climate chamber (Vötsch 4002 EMC), was used. The sintered samples were first polished by SiC sandpapers from 800 grit up to 4000 grit to remove any possible surface contamination and flatten the surface. Then, ∼200 nm of Au thin film was deposited onto both sides of the samples’ surfaces by using tabletop sputtering system to serve as ionic blocking electrodes. After the deposition of Au electrodes, samples were assembled in Swagelok cells by sandwiching between two nickel plates in Ar filled glove box. Desired bias voltage from 0.5 to 4.0 V was applied to the samples with a step size of 0.5 V. At each voltage, the current was monitored for 4 h to reach a steady DC current state for DC electronic conductivity calculations. Before the program went to the next bias voltage, the samples were set to open circuits for 30 min for relaxation. The measured I‐t curves are provided in Figures S6 and S7.

### 4.4 Microstructure Analysis

The microstructure was investigated using scanning electron microscopy (Zeiss GeminiSEM 560). Energy‐dispersive X‐ray spectroscopy (EDS) analysis was performed with an Ultim Max detector (100 mm^2^, Oxford Instruments). EDS maps were collected at an accelerating voltage of 10 kV and a working distance of 8.5 mm, with dimensions of 308 × 231 µm. EDS data were analyzed using Aztec software (Oxford Instruments). EDS point analysis was performed using the same working distance with a higher acceleration voltage of 15 kV. Raman spectroscopy was conducted with a WiTec Raman microscope, using solid‐state lasers with excitation wavelengths of 532 nm and 785 nm with a 600 grating per mm. The Raman microscope is integrated into the Zeiss GeminiSEM 560 SEM chamber, allowing for correlative SEM/EDS and Raman measurements. Raman maps were collected in high vacuum (∼ 10^−7^ mbar). The mapping was performed with a step size of 0.727 µm in both x and y directions, an integration time of 2 s, and a laser power of 7 mW. The total number of acquired spectra was 48,400. Raman data were analyzed using WiTec Project software (WiTec). EBSD measurements were performed on a FEI Quanta FEG 650 equipped with an EDAX Digiview 5 camera at 30 kV, spot size 6.5, working distance of 13 mm, and a step size of 300 nm.

### 4.5 First‐Principles Calculations

Density functional theory (DFT) calculations were conducted using the projector‐augmented‐wave method (PAW [40]) as implemented in the Vienna Ab‐Initio Simulation package (VASP [41, 42]). Electronic properties were analyzed through density of state calculations (DOS) with the strongly‐constrained and appropriately‐normed (SCAN [43]) exchange correlation functional. A plane‐wave kinetic energy of 520 eV and a 5 × 5 × 5 Monkhorst‐Pack [44] k‐point mesh were used, with an energy convergence threshold of 5 meV/atom. A denser k‐point mesh of 8 × 8 × 8 was employed for DOS calculations. Geometry optimizations for Co‐substituted LLZO structures were performed using electronic and ionic convergence criteria of 10^−5^ eV and 10^−2^ eV/Å, respectively. For DOS calculations, tighter convergence criteria of 10^−6^ eV and 10^−3^ eV/Å were adopted. A reduced cubic LLZO cell containing 96 total atoms (28 Li, 12 La, 8 Zr, and 48 O) with a cell dimension of 11.26 Å x 11.26 Å x 11.26 Å and angles of 109.47° was used. The defect formation energy of Co‐substitution was calculated as,

$$\;E_{f}=E_{\mathrm{total}}\;-E_{\mathrm{pristine}}-\sum_{i}n_{i}\mu_{i}$$

where E_total_ and E_pristine_ are the energies of the Co‐substituted and pristine LLZO supercells, respectively. n_i_ is the number of atoms of type i removed (<0) or added (>0) to maintain defect stoichiometry, and µ_i_ is the corresponding chemical potential. The chemical potential of Co was referenced to CoO and Co_3_O_4_, Li to bulk Li_2_O, Zr to bulk ZrO_2_, and La to bulk La_2_O_3_.

## Author Contributions

**Jehad Ahmed**: investigation, writing – original draft, writing – review and editing, validation, formal analysis, data curation, visualization. **Jordan A. Barr**: investigation, writing – original draft, writing – review and editing, visualization, validation, formal analysis, data curation. **Zhizhen Qin**: investigation, validation, writing – review and editing. **Anna Domgans**: investigation, validation, writing – review and editing. **Kwangnam Kim**: writing – review and editing, validation, project administration. **Chih‐Long Tsai**: supervision, project administration, formal analysis, conceptualization, writing – review and editing, investigation. **Roland Schierholz**: writing – review and editing, project administration, supervision, validation. **Julian Borowec**: writing – review and editing, investigation. **Brandon C. Wood**: project administration, writing – review and editing. **Liwen F. Wan**: project administration, writing – review and editing, validation. **Anna Windmüller**: writing – review and editing, validation, visualization, formal analysis. **Florian Hausen**: writing – review and editing, project administration, supervision. **Rüdiger Eichel**: writing – review and editing, funding acquisition, project administration, resources, supervision.

## Conflicts of Interest

The authors declare no conflicts of interest.

## Supporting information




**Supporting File**: advs78649‐sup‐0001‐SuppMat.docx.

## Data Availability

The data that support the findings of this study are available from the corresponding author upon reasonable request.
