# Supplementary material for: The Impact of Cobalt Substitution on the Electrochemical Properties of Cubic Li6.6La3Zr1.6Ta0.4O12 Solid Electrolyte for All‐Solid‐State Li‐Ion Batteries
Source: Adv Sci (Weinh). 2026 Nov 9:e78649. Online ahead of print. doi: 10.1002/advs.78649 (PMC13748886; doi:10.1002/advs.78649)
Supplement: Supplementary file 1 [file ADVS-9999-e78649-s001.docx]

**Supporting information**

**The impact of cobalt substitution on the electrochemical properties of cubic Li_6.6_La_3_Zr_1.6_Ta_0.4_O_12_ solid electrolyte for all-solid-state Li-ion batteries.**

Jehad Ahmed^1,2,*,†^, Jordan A. Barr^3,†^, Zhizhen Qin^1,2^, Anna Domgans^1,2^, Kwangnam Kim^4^, Chih-Long Tsai^1^, Roland Schierholz^1,*^, Julian Borowec^1,2^, Brandon C. Wood^4^, Liwen F. Wan^4,*^, Anna Windmüller^1^, Florian Hausen^1,2^, Rüdiger Eichel^1,2^

^1^Institute of Energy Technologies: Fundamental Electrochemistry (IET-1), Forschungszentrum Jülich GmbH, 52428, Jülich, Germany

^2^Institute of Physical Chemistry, RWTH Aachen University, 52074 Aachen, Germany

^3^School of Mechanical and Material Engineering, Washington State University, Pullman, WA 99164, USA

^4^Laboratory for Energy Applications for the Future (LEAF), Lawrence Livermore National Laboratory, Livermore, CA 94550, USA

^†^ These authors contributed equally to this work.

^*^Correspondence Email:[wan6@llnl.gov](mailto:wan6@llnl.gov); [j.ahmed@fz-juelich.de](mailto:j.ahmed@fz-juelich.de); [r.schierholz@fz-juelich.de](mailto:r.schierholz@fz-juelich.de)

Strocka

a [Å]

| x (Co) | Co2+ on Li | Co3+ on Li | Co3+ on Zr | Co3+ on La |
| --- | --- | --- | --- | --- |
| 0 | 12.933 | 12.933 | 12.933 | 12.933 |
| 0.005 | 12.933 | 12.933 | 12.9327 | 12.9313 |
| 0.01 | 12.933 | 12.9331 | 12.9323 | 12.9296 |
| 0.03 | 12.9329 | 12.9332 | 12.931 | 12.9228 |
| 0.05 | 12.9329 | 12.9333 | 12.9296 | 12.9161 |
| 0.1 | 12.9327 | 12.9335 | 12.9264 | 12.8997 |
| 0.16 | 12.9326 | 12.9339 | 12.9227 | 12.8808 |

Figure S1: Lattice parameter of Co-substituted LLZTO calculated with the empirical formula of Strocka et al. [3] for Co^3+^ on the Li, Zr, and La sites and for Co^2+^ on the Li site, as a function of the Co content x.

Empirical formulas exist in the literature that allow the lattice parameter of garnets to be calculated from the ionic radii [1] of the ions on the different structural sites.[2] We tested them and found that the formula of Strocka et al. [3] gives the best agreement with the crystallographic data (.cif files) of Kataoka et al. [4] for LLZTO. Figure S1 shows that for Co^2+^ as well as Co^3+^ on the Li site essentially no change of the lattice parameter is expected. Even for Co^3+^ on the Zr site the change is small for x ≤ 0.1.

The grain size and crystallographic orientations of the Argon (Ar)- and air-sintered samples were investigated using EBSD. Prior to the EBSD measurements, the samples were mechanically polished down to a final step of 0.05 µm to obtain a surface suitable for indexing. EBSD data were acquired at an acceleration voltage of 30 kV using an EDAX DigiView EBSD camera, and the datasets were processed using OIM Analysis software (v8.6). The results are summarized in Figure S2, where the EBSD maps and the corresponding grain size distributions show that both processing conditions yield comparable microstructures: the grain diameters predominantly fall in the 4–5 µm range. In addition, grain orientation maps do not indicate any preferred texture, suggesting a random crystallographic orientation distribution for both air- and Ar-sintered samples.

Figure S2: (a) EBSD orientation maps of the air- and argon-sintered samples. (b,c) Grain size distributions extracted from the EBSD data for Co contents x = 0.005, 0.03, and 0.1, shown for the air-sintered samples in (b) and the corresponding Ar-sintered samples in (c).

The pore segmentation was performed to quantitatively compare microstructural differences between Ar- and air-sintered samples. In particular, the analysis highlights that Ar-sintered samples exhibit smaller pores that are frequently interconnected into a pore network, whereas air-sintered samples show fewer but larger pores. Quantitatively, the air-sintered samples show an average mean pore area of 69.59 ± 7.26 µm², while the Ar-sintered samples exhibit a markedly lower average mean pore area of 31.32 ± 6.71 µm². A global threshold was set using Otsu’s method [5]. Pixels darker than the threshold were classified as pores, giving a binary porosity mask. Porosity was calculated as the fraction of pore pixels, and pore sizes were obtained by labeling connected pores, removing very small speckles.

Figure S3: Representative electron micrographs of (a) the air-sintered sample and (b) the Ar-sintered sample. The corresponding pore segmentation mask is shown directly beneath the electron image, where the segmented regions identify the pore phase used for quantitative porosity and pore-mean area analysis.

Figure S4: Nyquist plots and fits of LLZTO sintered in (a) air, (b) Ar and (c) Co_0.1_:LLZTO in air at 970 °C.

Figure S5: DFT calculated density of states (DOS) for the lowest-energy Co substitution configurations at the different cation sites in LLZO, using the SCAN functional.

Figure S6: I-t curves for air-sintered Co_x_:LLZTO.

Figure S7: I-t curves for Ar-sintered Co_x_:LLZTO.

Figure S8: DC electronic conductivity at 25 °C and 4 V (left axis) and LiCoO₂ weight fraction from Rietveld refinement (right axis) of the air-sintered Co_x:LLZTO samples as a function of the nominal Co content x.

Figure S9: Co content of the LLZTO grain interiors from SEM-EDS point analysis (filled symbols, left axis; ten measurement points per sample) together with (a) the total conductivity and (b) the electronic conductivity at 25 °C and 4 V (open symbols, right axis).

**Table S1.** (a) Charge-balanced compositions of Co-substituted LLZTO (Li₆.₆La₃Zr₁.₆Ta₀.₄O₁₂), showing the possible valence states and positions of Co, and the cubic LLZO unit cell used in the DFT calculations (one Co atom per four formula units, x = 0.25). (b) The expected Co content upon complete incorporation, calculated based on all atoms as well as the elements detectable by EDS (excluding Li), as a function of the nominal x-value.

(a) Compositions

| Co valence / site | Composition (experimental, LLZTO) | Charge compensation per Co | DFT cell (cubic LLZO, x = 0.25) |
| --- | --- | --- | --- |
| Co^2+^ on Li | Li_6.6−2x_Co_x_La_3_Zr_1.6_Ta_0.4_O_12_ | 1 Li^+^ replaced + 1 Li vacancy | Li_6.5_Co_0.25_La_3_Zr_2_O_12_ |
| Co^3+^ on Li | Li_6.6−3x_Co_x_La_3_Zr_1.6_Ta_0.4_O_12_ | 1 Li^+^ replaced + 2 Li vacancies | Li_6.25_Co_0.25_La_3_Zr_2_O_12_ |
| Co^3+^ on Zr | Li_6.6+x_Co_x_La_3_Zr_1.6−x_Ta_0.4_O_12_ | 1 Li interstitial | Li_7.25_Co_0.25_La_3_Zr_1.75_O_12_ |
| Co^3+^ on La | Li_6.6_Co_x_La_3−x_Zr_1.6_Ta_0.4_O_12_ | none | Li_7_Co_0.25_La_2.75_Zr_2_O_12_ |
| Co^3+^ on Ta | Li_6.6+2x_Co_x_La_3_Zr_1.6_Ta_0.4−x_O_12_ | 2 Li interstitials | – |

(b) Expected Co content for complete incorporation

| nominal x | 0.005 | 0.01 | 0.03 | 0.05 | 0.1 | 0.25 (DFT, LLZO) |
| --- | --- | --- | --- | --- | --- | --- |
| Co (at%), all atoms | 0.02 | 0.04 | 0.13 | 0.21 | 0.42–0.43 | 1.03–1.06 |
| Co (at%), without Li (EDS basis) | 0.03 | 0.06 | 0.18 | 0.29 | 0.59 | 1.45–1.47 |

**References**

1. Shannon, R.D., Revised effective ionic radii and systematic studies of interatomic distances in halides and chalcogenides. Acta Crystallographica Section A, 1976. 32(5): p. 751–767 (database: http://abulafia.mt.ic.ac.uk/shannon/ptable.php).
2. Drazin, J.W. and R.S. Hay, *Refractive index and lattice parameter prediction for garnets (A3B2C3X12).* Journal of the American Ceramic Society, 2023. **106**(6): p. 3853–3866.
3. Strocka, B., P. Holst, and W. Tolksdorf, *An empirical formula for the calculation of lattice constants of oxide garnets based on substituted yttrium-and gadolinium-iron garnets.* Philips Journal of Research, 1978. **33**(3/4): p. 166–202.
4. Kataoka, K. and J. Akimoto, *Lithium-ion conductivity and crystal structure of garnet-type solid electrolyte Li_7−x_La_3_Zr_2−x_Ta_x_O_12_ using single-crystal. Journal of the Ceramic Society of Japan, 2019. 127(8): p. 521–526.*
5. Otsu, N., A threshold selection method from gray-level histograms. IEEE Transactions on Systems, Man, and Cybernetics, 1979. 9(1): p. 62-66. https://doi.org/10.1109/TSMC.1979.4310076
